# Assessment of Knowledge Regarding Cataract Among Saudi Adult Population in Assir Region, Saudi Arabia

**DOI:** 10.7759/cureus.32703

**Published:** 2022-12-19

**Authors:** Waleed Aldhabaan, Majdoleen A Abdulrahman, Mohammed Y Asiri, Mohanad Q Alshabab, Majed Y Alshahrani, Ghadeer R Alnakhli, Faris Alasmari, Amnah Alharthi, Ahmed S AL Zomia

**Affiliations:** 1 Ophthalmology, King Khalid University, Abha, SAU; 2 Medicine, King Khalid University, Abha, SAU; 3 Medicine, Aseer Hospital Center, Abha, SAU; 4 College of Medicine, King Khalid University, Abha, SAU

**Keywords:** cataract risk factor, cataract treatment, cataract complications, cataract extraction, knowledge, s: cataract surgery, cataract, cataract patients

## Abstract

Introduction: Cataract is the most prevalent age-related eye disease and the most curable cause of adult visual impairment. The aim of this study was to assess the cataract disease rate and knowledge regarding its definition, symptoms, risk factors, prognosis, and treatment.

Methods: A community-based cross-sectional study of 600 randomly selected people aged 18 and up was conducted from May 2022 to August 2022, among adults. Participants were given an online survey via social media that included items testing their knowledge of cataracts.

Results: Study participants had a 3.4% cataract previous exposure rate. Four hundred forty-eight (75.9%) participants had good knowledge about cataracts. Participants with higher educational levels were more likely to correctly answer questions about different aspects of knowledge about cataracts.

Conclusion: Future studies should focus on improving awareness about cataracts. Risk factors, complications, treatment options, complications of cataract surgery, and regular follow-ups should be explained to patients. Through routine eye checks, early detection and treatment of this condition will be better understood.

## Introduction

Vision impairment and age-related eye illnesses have a negative impact on economic and educational opportunities, as well as a lower quality of life and an increased chance of death as in cataracts [1]. According to the World Health Organization, there are 180 million visually impaired people worldwide, with 40-45 million of them unable to walk unaided. Cataracts are thought to be the cause of 46% of these occurrences [2]. A cataract is the primary cause of blindness worldwide [3]. A cataract is also the most expensive eye illness requiring treatment in the United States, accounting for more than 60% of yearly Medicare eye-related spending [4] and also could be related to diabetes mellitus, cardiovascular disease, and a lot of risk factors [5].

A cataract is the most prevalent age-related disease of the eye [6]. A cataract is an opacity of the lens of the eye, which may lead to increased light scattering [7]. It is also the most curable cause of adult visual impairment [6]. It is a multifactorial disease that could have a genetic, socio-demographic, behavioral, or environmental basis [7]. Although it is probably true that these factors interact with each other, age remains the single most important risk factor for cataracts [7]. It develops slowly to cause loss of vision and can render the person completely blind if it is left untreated [8].

Cataract has a consequent impact on the individual, family, community, and nation in terms of visual disability. It reduces the quality of life, independence, and social interaction [9]. Despite being the leading cause of preventable and treatable blindness, a lack of knowledge about the disease and its treatment options remains a major barrier to reducing cataract-related blindness in developing countries [10]. The proportion of blindness because of cataracts among all eye disease levels ranges from 5% in developed countries to 50% or more in low- and middle-income areas of western and eastern sub-Saharan Africa (5.1%) and South Asia [10]. In the southwest of Saudi Arabia, 0.7% of people are blind, while 10.9% have some form of vision impairment. 52.6% of blindness and 20.6% of vision impairment are caused by cataracts [6]. Knowledge about cataracts is the most vital aspect for delaying the incidence of cataracts, initiating regular eye check-ups, and instituting timely intervention. As a result, the disease's burden is reduced [11]. Several previous studies revealed that there was a gap in data concerning cataracts in developing and some developed countries. The research also considered age, literacy, residency, marital status, previous exposure to eye care services, and other socio-economic variables as determinants of knowledge concerning cataracts [11]. It is essential that public education messages be spread concerning all eye diseases and conditions that affect older adults. The importance of public health education is to ensure that older adults have health literacy [6].

Even after correcting for recognized risk variables, less education and poorer income are associated with greater morbidity and mortality from a variety of diseases. These associations have been linked to a lack of utilization of healthcare resources, high-risk behaviors, exposure to hazardous jobs or unpleasant family settings, and poor diet [12].

Eye care programs should focus on preventing visual impairment caused by refractive errors, screening for incurable chronic eye diseases, and promoting health education to raise awareness of cataracts [13]. Hence, we conducted a survey among the general population in the Assir region to assess their level of awareness about cataract causes and risk factors. Our specific objectives have been to assess the prevalence of patients who have been diagnosed with cataracts, to assess the degree of knowledge about cataract causes and risk factors, and to measure factors related to the care, prevention, and management of cataracts among individuals living in the Assir region of Saudi Arabia.

## Materials and methods

A cross-sectional study was undertaken from May 2022 to August 2022 to assess the knowledge of cataracts among the population living in the Assir region, Saudi Arabia. The sample size was 600 individuals from adult people of both genders who are Saudi living in the Assir region. The Ethical Committee of Scientific Research of King Khalid University accepted the study with approval number (ECM#2022-2116)- (HAPO-06-B-001).

It includes all adult participants in Assir regions. Including criteria involve all Saudi adult population over 18 years old who lives in the Assir region. Exclusion criteria involve anyone who lives outside the Assir region. Non-Saudi people 17 years old or less. The questions include adult bio demographic data, prevalence, and knowledge of cataracts, risk, and complication of cataracts, and finally awareness regarding the treatment.

Data sources/measurement

It includes all adult Saudi population by using digital survey and distribution using social media, the survey consists of questions to assess the knowledge of cataracts among the Saudi adult population. data were collected using a self-administered online questionnaire containing questions for adult age, education, occupation, history of having cataracts, and awareness of the disease, risk factors, complications, and treatment. The questionnaire was developed by the researchers after an intensive literature review and expert consultation. It was reviewed by experts independently and any suggested correction was applied. The final questionnaire was uploaded online in Arabic language using social media Twitter, Instagram, and WhatsApp platforms by the researchers and their friends and relatives to reach more people during the period from May 25, 2022 to August 11, 2022 till the sample size was adequate, the adult who live out of Aseer region, those who were less than 18 years were excluded.

Statistical methods

The data were collected and verified manually, then it was coded before entering it into the computers, the statistical package for social science (SPSS) software version 22.0 was used for data entry and analysis.

## Results

In this cross-sectional study, 600 participants were included. Ten patients refused to participate. The study enrolled 590 participants; 54.9% were male and 66.8% were 18 to 29 years old. Half (50.0%) were married. Bachelor's/diploma was the last educational qualification with 79.7%. Employed respondents represented 40.8% of the sample and students 34.4%. A total of 3.4% reported a previous clinical diagnosis of cataracts. Table 1 displays the study sample's baseline characteristics.

**Table 1:** Baseline characteristics of the study sample (n = 590)

Variables	Frequency
Sex	
Male	324
Female	266
Age group (years)	
18-29	394
30-39	73
40-49	76
≥50	34
Marital status	
Single	295
Married	295
Employment status	
Unemployed	122
Employed	241
Student	203
Retired	24
Previous diagnosis of cataract	
No	570
Yes	20

Table 2 shows that three-quarters of subjects (75.9%) were aware of cataracts as an ophthalmologic health condition. The correct definition of cataract (i.e., the opacity of the eye lens) was identified by 47.6% of subjects. While 88.6% knew that aging is a major risk factor for cataracts, 42.4% did not know that a family history of cataracts increases the risk of developing the condition. With respect to complications of cataracts, more than one-third (64.1%) did know that blindness is one of the cataract complications (Table 2).

**Table 2:** Knowledge about the definition, risks, and complications of cataract (n = 590).

Variables	Frequency			Percent
Do you know an eye disease called cataract?				
No		142	24.1%	
Yes		448	75.9%	
Definition of cataract				
I do not know/no		71	12.0%	
The presence of white color on the eye lens		164	27.8%	
Opacity of eye lens		281	47.6%	
Decreased visual acuity at night		73	12.4%	
The absence of eye lens		1	.2%	
Family history increases the risk to develop cataract				
No		250	42.4%	
Yes		340	57.6%	
Aging increases the risk to develop cataract				
No		67	11.4%	
Yes		523	88.6%	
Cataract might lead to blindness				
No		212	35.9%	
Yes		378	64.1%	

**Figure 1:**
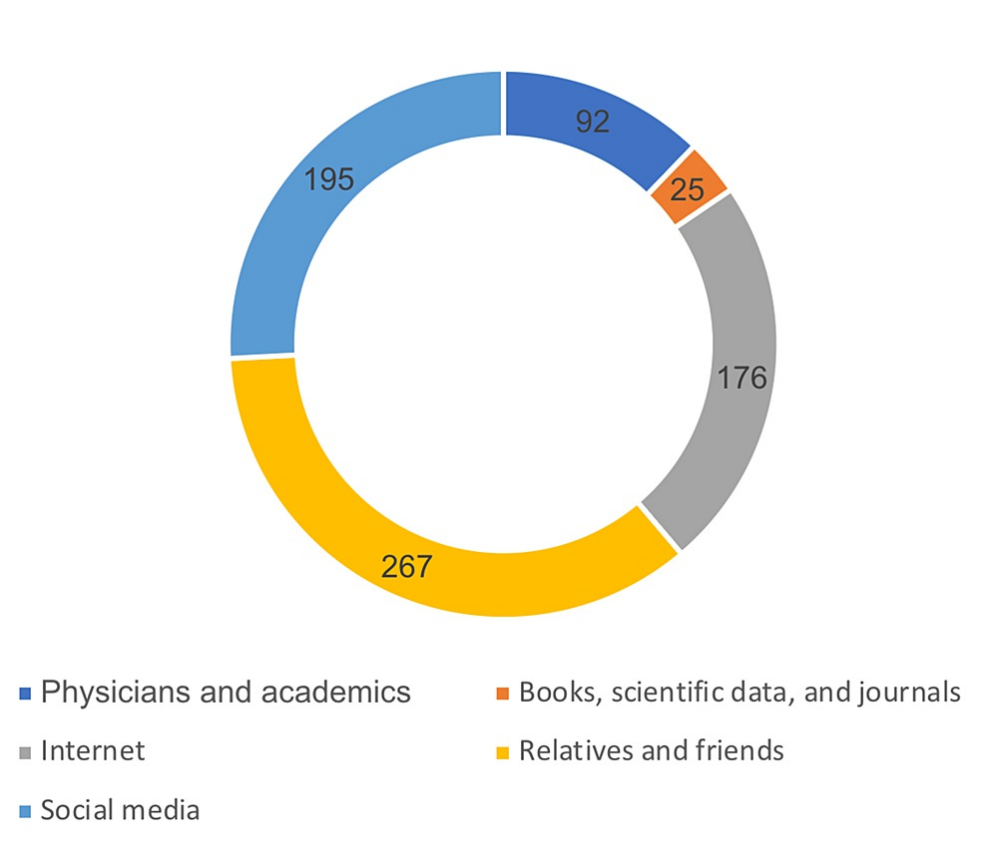
Source of knowledge about cataracts in the study population (n = 590)

Figure 1 summarizes sources of knowledge about cataracts among the study subjects, showing that relatives and friends were the most frequent source of cataract information (n=267), followed by social media (n=195) and the internet (n=176).

**Figure 2:**
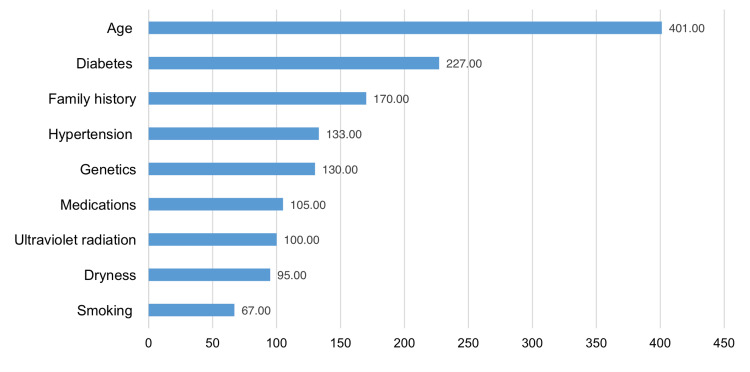
Knowledge about cataract symptoms among the study population (N = 590)

Figure 2 illustrates knowledge about cataract risk factors among the study subjects, showing that age (n=401), diabetes (n=227), and family history (n=170) were the most commonly cited risk factors for cataracts.

**Figure 3:**
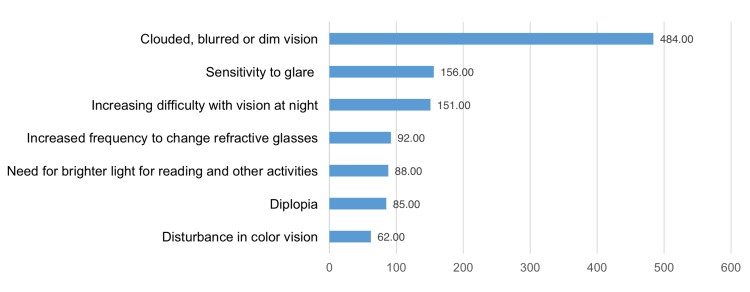
Knowledge about symptoms of cataracts in the study population (N = 590)

Figure 3 illustrates knowledge about cataract symptoms among the study subjects, showing that clouded, blurred, or dim vision (n=484), sensitivity to glare (n=156), and visual difficulties (n=151) were the most commonly cited symptoms of cataracts.

As shown in Table 3, 83.9% thought surgical corrections is an intervention to treat cataracts, while only 6.8% thought that cataracts could be treated medically regardless of lens maturity and degree of visual acuity. In addition, 79.8% agreed that cataract is treated surgically regardless of visual acuity, while more than half (56.4%) knew that surgical intervention is the only treatment option once the cataract has become mature (Table 2).

**Table 3:** Knowledge about the treatment of cataracts (n = 590).

Variable	Frequency	Percentage
Treatment modalities		
I do not know/no	55	9.3
Surgical	495	83.9
Pharmacological	40	6.8
Cataract is treated surgically when it affects vision		
I do not know/no	119	20.2
Yes	471	79.8
Cataract is treated surgically when the lens is mature		
I do not know/no	332	56.3
Yes	258	43.7

Table 4 shows that knowledge about cataracts was significantly associated with the level of education with respect to awareness of cataracts as an ophthalmologic condition (p<0.001), cataract complications (p<0.001), as well as heritability (p<.001) aging (p<0.001) as risk factors for the condition. Also, there was a significant difference in education regarding knowledge about the surgical treatment of cataracts (p<0.05). These findings indicate that subjects with higher educational levels were more likely to correctly answer questions about different aspects of knowledge about cataracts. However, education level was not associated with knowledge about surgery as a treatment modality for cataracts (p<0.05) (Table 4).

**Table 4:** The relationship between education and knowledge about cataracts (n = 590).

Variables	High school/lower	Bachelor/diploma	Master/doctorate	X^2^	P-value
Awareness of cataract as an ophthalmologic condition	55.8%	79.1%	92.0%	27.284	< 0.001>
Cataract might lead to blindness	52.6%	66.0%	72.0%	6.809	< 0.001>
Heritability of cataract	38.9%	61.3%	60.0%	16.197	< 0.001>
Aging as a risk factor for cataract	72.6%	91.5%	96.0%	29.321	< 0.001>
Surgery as a treatment modality of cataract	75.8%	85.1%	92.0%	7.546	0.110
Cataract is treated surgically when it affects vision	67.4%	82.6%	76.0%	11.555	0.003
Cataract is treated surgically when the lens is mature	30.5%	46.2%	48.0%	8.053	0.018

## Discussion

This study aimed to assess the cataract disease rate and knowledge regarding its definition, symptoms, risk factors, prognosis, and treatment among adults living in the Assir region, Saudi Arabia. Study participants had a 3.4% cataract previous exposure rate, this was close to other studies [6,14] and three-quarters of the participants knew about cataract disease. Cataract was defined correctly by almost half of our participants (i.e., an opacity of the eye lens). This was in line with the previous study by Alammar et al. [6] among the public in the Kingdom of Saudi Arabia (KSA) and in contrast with Magliyah et al.'s study among the adult population in Makkah City, KSA, which found that 72.4% of their participants did not know that cataract is an increase in the opacity of the eye lens [14].

In our study and Alammar et al.'s [14] study, more than half of the participants knew that family history and aging are risk factors for cataracts, and it leads to blinding. On the other hand, Magliyah et al. found that more than 75% of participants did not know that the incidence of cataracts increases with positive family history and age and did not know that cataracts can lead to blindness [14].

The present study confirmed the findings of Alammar et al. [6] and Alamri et al. [15] that participants considered hypertension and diabetes mellitus to be important cataract risk factors.

The majority of participants in this study recommended surgery as a treatment for cataracts. This is similar to the results reported by Alamri et al. [15]. However, Magliyah et al. [6] found that almost two-thirds of the participants did not know how cataracts are treated when they impair vision.

Participants with higher educational levels were more likely to correctly answer questions about different aspects of knowledge about cataracts and that was in line with previous studies [6,14]. However, the differences between other studies’ results and our study results, especially the studies that showed poor knowledge [14] could be due to deficiencies in public health education regarding eye disease and knowledge gaps that healthcare providers provide in their health education programs. In cooperation with King Khaled Eye Specialist Hospital, the Saudi Ophthalmology Society made a significant effort of organizing special events to raise public awareness about cataracts and other eye diseases [16].

## Conclusions

The adult Saudi population in the Assir region have good knowledge about cataract. Future studies should focus on improving awareness regarding cataracts. The patients should receive education about risk factors, complications, treatment options for the cataract disease, complications of the surgery, and the regular need for a follow-up. As a result, routine eye checks will be better understood for the early detection and treatment of these conditions.
